# A Case of Comorbid PTSD and Posttraumatic OCD Treated with Sertraline-Aripiprazole Augmentation

**DOI:** 10.1155/2020/2616492

**Published:** 2020-01-27

**Authors:** Rodolfo Rossi, Cinzia Niolu, Alberto Siracusano, Alessandro Rossi, Giorgio Di Lorenzo

**Affiliations:** ^1^Chair of Psychiatry, Department of Systems Medicine, University of Rome Tor Vergata, Rome, Italy; ^2^Psychiatry and Clinical Psychology Unit, Fondazione Policlinico Tor Vergata, Rome, Italy; ^3^Department of Applied Clinical Sciences and Biotechnologies, University of L'Aquila, Via Vetoio, 67100 L'Aquila, Italy

## Abstract

Several studies report on traumatic history in Obsessive Compulsive Disorder (OCD) and comorbidity between Posttraumatic Stress Disorder (PTSD) and OCD. First-line pharmacological treatments for both OCD and PTSD are primarily based on antidepressants, including Selective Serotonin Reuptake Inhibitors (SSRIs) and Serotonin-Noradrenaline Reuptake Inhibitors (SNRI) such as Venlafaxine for PTSD. Second-Generation Antipsychotic (SGA) augmentation has shown good outcomes for nonresponsive OCD cases. However, evidence on the use of SGA in PTSD is more limited. In the present paper, we report on comorbid OCD-PTSD successfully treated with aripiprazole augmentation of sertraline. Shared psychopathological and pharmacological aspects of the disorders are discussed.

## 1. Introduction

Several studies report on traumatic history in Obsessive Compulsive Disorder (OCD) [1] and comorbidity between Posttraumatic Stress Disorder (PTSD) and OCD. It is still debated whether OCD occurring after traumatic exposure is a manifestation related to PTSD or represents an independent comorbidity, requiring an independent intervention. However, posttraumatic OCD displays a different clinical phenotype, with more severe obsessive compulsive symptoms, and a more severe general clinical picture, including depression, anxiety, and suicidality [2].

Prevalence of OCD among individuals with PTSD is about 30%, compared to 1% in the general population [3], with posttraumatic and OC symptoms showing even a larger association [4, 5]. Comorbidity between OCD and PTSD results in complex clinical presentations, problematic management, and poor outcome.

Under a psychopathological perspective, OCD and PTSD share at least three clusters of symptoms: unwanted intrusive thoughts, memories, or images; repetitive behaviours aimed at reducing distress; and avoidance of stimuli associated with unwanted thoughts which are often centred around themes of guilt and responsibility for harm [5]. Alongside with formal overlaps between OCD and PTSD symptoms, some content differences are worth noting. Firstly, in PTSD, intrusive thoughts are generally memories of past events, while in OCD, intrusions are related to future potential threats. Secondly, PTSD involves avoidance of internal or external reminders of painful memories, while OCD involves avoidance of feared potential consequences. Finally, dissociative symptoms, core features of stress-related disorders [6, 7], have been shown to be associated with OCD as well. In particular, some studies have highlighted that dissociative symptoms in trauma-related OCD are associated with a more severe clinical picture as well as treatment resistance [8, 9].

Neurobiological studies have highlighted the different roles of the main neurotransmitters and other signaling molecules. Among neurotransmitters, serotonin, norepinephrine, and dopamine have been involved in the pathophysiology of both OCD and PTSD. Among nonneurotransmitter molecules, BDNF has been addressed as a neuromodulator of trauma-related responses in both OCD and PTSD [10, 11]. Altered levels of BDNF have also been related to impulsiveness in PTSD [12] and could be therefore considered a link between OCD and PTSD. However, evidence of the role of BDNF in OCD is still weak [13].

Psychological exposure-based interventions are considered the mainstay first-line treatment for both PTSD and OCD [14, 15]. Despite strong evidence supporting psychological interventions in PTSD and OCD, in everyday practice, psychological interventions may not be feasible and pharmacological interventions may be the only viable option. However, evidence supporting pharmacological treatment options for comorbid PTSD and OCD remains elusive.

First-line pharmacological treatments for both OCD and PTSD are primarily based on antidepressants, including Selective Serotonin Reuptake Inhibitors (SSRIs) [16–18] and Serotonin-Noradrenaline Reuptake Inhibitors (SNRI) such as Venlafaxine for PTSD.

Second-Generation Antipsychotic (SGA) augmentation has shown good outcomes for nonresponsive OCD cases [19, 20]. In particular, aripiprazole, risperidone, or paliperidone augmentation of SSRI agents has shown good outcomes [21].

The efficacy of SGA in PTSD is less clear. Risperidone and olanzapine have shown good efficacy in PTSD, with low level of evidence. Evidence on aripiprazole use in PTSD is still weak. A recent systematic review identified 6 studies of which only one was an RCT [22]. However, aripiprazole showed good efficacy in controlling PTSD symptoms.

In the present paper, we report on comorbid OCD-PTSD. Some common psychopathological aspects will be discussed, as well as a successful treatment with aripiprazole augmentation of sertraline.

## 2. Case Presentation

M.C., a 35-year-old male, presented to our Community Mental Health Service for a chief complaint of irritable mood and insomnia following a severe car accident.

Three months before, M.C. was involved in a car accident on a highway. The patient remained unharmed, while the driver of the other vehicle died soon after the impact. The patient witnessed the rescue and death of the other person. Although he reported that the incident was caused by the other person, he would have to face a trial during the following months; after that, the victim's relatives had sued him.

On the first diagnostic interview, the patient reported typical PTSD symptoms, of which flashbacks were experienced as the most disturbing, followed by avoidance of external reminders of the accident and hyperarousal with panic attacks. Numbing was the least frequent and disturbing symptom. The patient reported also depressed mood with irritability and rage outbursts. Thought content was characterized by themes of guilt for his parents who had to help him out with subsequent economic difficulties. No psychotic symptoms or perceptual abnormalities were elicited. The sleep cycle was disturbed with frequent nightmares regarding the accident.

The patient was experiencing moderate functional impairment, as he had to drive for work, with enormous difficulties due to continuous flashbacks and hyperarousal. Moreover, frequent rage outbursts were occurring at work.

The patient denied any illicit drug or alcohol abuse, and there was no history suggestive of alcohol or illicit drug abuse. There was no family history of mental or physical illness. His socioeducational level was low: he came from a poorer region of Italy, having to move some years before to look for a job. He worked as a constructor and had attended only the first two years of high school without graduating.

Protective resilience factors were scarce: the patient had a very limited social network, with his family living in another region in Italy.

The Davidson Trauma Scale (DTS) score on the first evaluation was 75/136.

The patient was offered both psychological and pharmacological treatments. After declining psychological treatment, an initial treatment with sertraline 50 mg was offered and reevaluation was scheduled after 3 weeks.

During the following 3 months, sertraline was progressively titrated up to 150 mg with only minor clinical improvement. Flashbacks and hyperarousal were relatively reduced; the overall clinical picture was still unsatisfactory due to low and irritable mood.

Given the lack of response, a diagnostic revision was carried out using a SCID-5 structured diagnostic interview. Previously undetected obsessive compulsive symptoms were found, fulfilling the criteria for an OCD diagnosis. According to the patient's reports, OCD had begun soon after the accident, together with posttraumatic symptoms. The main compulsions were of checking type (door lock, car door, and gas stoves). During the following interviews, it emerged that compulsions were psychologically related to the trauma and guilt ideation. Compulsions in fact were associated with fear of harming economically his parents if he had put himself into further economic difficulties, for example, having his car stolen or his house burned down. After a brief psychoeducational intervention on OCD, off-label aripiprazole 5 mg was added and titrated up to 15 mg in the following 2 weeks.

On a total of sertraline 200 mg and aripiprazole 15 mg, subjective clinical response took over, and over a period of 2 months, both PTSD and OCD were in full and stable remission, with DTS falling to 13/136. During the follow-up interviews, the patient reported a constant reduction in flashback frequency and associated psychological distress. Progressive exposure to external reminders of the car accident gradually restored his working capacity. Compulsions were managed using the basic Exposure Response Prevention (ERP) technique: the patient reported only minimal effort in resisting to the urge, while before initiation of aripiprazole, resistance was practically impossible for him. Residual insomnia was treated with 25 to 50 mg of trazodone as needed.

Bimonthly visits were scheduled as a follow-up, and the patient was discharged after 1 year, on sertraline 50 mg and aripiprazole 5 mg.

## 3. Discussion

This case demonstrates a tight connection between OCD and PTSD under a psychological and pharmacological perspective. In recent years, comorbidity among mental disorders has been addressed using a network approach to psychopathology, which conceptualizes disorders resulting from the causal interplay between symptoms [23]. Although no direct network-based investigation of comorbid PTSD-OCD is available, intrusive thoughts and imagery have been identified as a central feature of PTSD [24] within a network paradigm. Moreover, intrusive memories of remote events have been identified in about one-third of patients with OCD [25]. The singularity in this patient is that intrusive thoughts that characterized and maintained both OCD and PTSD were posttraumatic, with both OCD and PTSD starting soon after the traumatic event.

Under a cognitive thematic approach, both OCD and PTSD were associated with themes of guilt and responsibility of harm. This case illustrates clearly how the negative appraisal of the consequences of a traumatic event may trigger a responsibility of harm theme, culminating in a full-blown OCD. Depressed mood was associated with a negative self-image that could have maintained both OCD and PTSD symptoms.

Aripiprazole is effective either as monotherapy or as adjunct for both PTSD [22] and OCD [19], although evidence of monotherapy in OCD is modest [26].

The main effect exerted by aripiprazole in this case was to relieve intrusive thoughts, mitigating both OCD and PTSD symptoms. One putative mechanism for the effectiveness of aripiprazole in both PTSD and OCD may be related to the modulatory effects that antidopaminergic drugs have on the cellular synaptic plasticity and memory consolidation [27]. This clinical observation suggests that dopaminergic modulation of intrusive thoughts may be a research topic and therapeutic target of interest cutting across several diagnostic categories, including PTSD and OCD. On the other hand, aripiprazole has been suggested to act on PTSD symptoms via its serotoninergic activity on 5-HT1a receptors [22]. This effect, which is potentiated synergistically with SSRIs such as sertraline [28], could ameliorate depressive symptoms in the first place, with the improvement on both OCD and PTSD symptoms being potentially mediated by the effects on depressed mood.

This case report opens a number of experimental questions. Firstly, it fosters further research on comorbidity in trauma-exposed subjects and on the interplay among several psychopathological domains, possibly using a network approach. Secondly, it opens a question on how different pharmacological interventions act on the symptom network and on which is the hierarchy within a symptom network relevant to pharmacological treatments.
